# Pyrolysis temperature effects of tomato stems biochar on leaching dynamics of ammonium, nitrate, and dissolved organic carbon in sandy soil

**DOI:** 10.1038/s41598-026-41017-0

**Published:** 2026-03-17

**Authors:** Amer Eisa Amer, Mohamed Ali El-Desoky, Abu El-Eyuoon Abu Zied Amin, Hosny Mubarak Farrag

**Affiliations:** 1 Soils and Water Department, Faculty of Agriculture, Qena University, Qena, Egypt; 2 Soils and Water Department, Faculty of Agriculture, Assiut University, P.O. Box: 71526, Assiut, Egypt

**Keywords:** Ammonium, Dissolved organic carbon, Nitrate, Pyrolysis temperature, Sandy soil, Tomato stems biochar, Ecology, Ecology, Environmental sciences

## Abstract

The study objectives are to examine the effect of doses of tomato stems biochar (TSB) produced at different pyrolysis temperatures (250, 400, and 600 °C) on the leaching of nitrate, ammonium, and dissolved organic carbon, as well as quality indicators of sandy soil. The column experiment was including these treatments; control (no biochar added), 1% TSB250, 2.5% TSB250, 5% TSB250, 1% TSB400, 2.5% TSB400, 5% TSB400, 1% TSB600, 2.5% TSB600, and 5% TSB600. Each plastic column was filled with 1 kg of sandy soil. Tomato stems biochar was applied at three doses (1%, 2.5%, and 5% w/w). Soil available nitrogen increased significantly relative to the control treatment by 9.50%, 31.69%, 46.71%, 69.07%, 15.24%, 37.43%, and 75.57% under applying 1% TSB250, 1% TSB400, 2.5% TSB400, 5% TSB400, 1% TSB600, 2.5% TSB600, and 5% TSB600 treatments, respectively. Results showed significant decreases in cumulative leached ammonium over the control treatment by 20.77%, 27.04%, 37.09%, 34.04%, 40.43%, 48.61%, 18.26%, 25.26%, and 32.94% for 1% TSB250, 2.5% TSB250, 5% TSB250, 1% TSB400, 2.5% TSB400, 5% TSB400, 1% TSB600, 2.5% TSB600, and 5% TSB600 treatments, respectively. The amount of cumulative leached nitrate decreased significantly relative to the control treatment by 8.27%, 8.56%, 8.91%, 8.61%, 8.42%, 8.66%, 28.37%, 31.63%, and 34.40% for 1% TSB250, 2.5% TSB250, 5% TSB250, 1% TSB400, 2.5% TSB400, 5% TSB400, 1% TSB600, 2.5% TSB600, and 5% TSB600 treatments, respectively. The effectiveness of biochar treatments in reducing the cumulative leaching of ammonium decreased in the order TSB400 > TSB250 > TSB600. However, the effectiveness of biochar treatments on the cumulative leaching nitrate was in the order of TSB600 > TSB400 ≈ TSB250. Applying TSB at all pyrolysis temperatures and levels in sandy soil led to a significant increase in the cumulative leaching of dissolved organic carbon compared to the control treatment. Utilizing tomato stems biochar as a soil amendment is a promising strategy for significantly enhancing the quality indicators of sandy soil and reducing the leaching of ammonium and nitrate. This would reduce the loss of nitrogen fertilizers added to the soil and preserve groundwater from pollution.

## Introduction

Human activities have caused climate change, environmental pollution, and unsustainable agriculture, fertilizer leaching, and a deficiency of food security worldwide^1^. The world increasingly relies on fertilizers, especially industrial nitrogen fertilizers, to meet the needs of a growing population and increase crop yields^2^. Increased leaching of nitrate in soil water is a serious environmental concern, where nitrate (NO_3_^−^) is a major pollutant of groundwater worldwide^3^. Ammonium can transform into nitrate through the process of nitrification by soil microorganisms^4^. High levels of nitrate that exceed 10 mg L^−1^ in water can cause many environmental and health issues, like methemoglobinemia or “blue baby syndrome”, which affects infants^5^. Nitrate also enters the composition of N-nitroso compounds, most of which are carcinogens^6^. The amount of nitrogen (particularly nitrate) that might be lost by leaching could range from 1 to 35% of the total N fertilizer application (equivalent to 15–45 kg ha^−1^)^7^, leading to low N fertilizer-use efficiency; therefore, its cost increases^8^. Especially sandy soil, their limited water retention is due to their high sand content^9^. This soil is easily leached of nutrients such as nitrogen (N) as NO_3_^−^ and carbon (C) as dissolved organic carbon (DOC). Therefore, it is necessary to find an effective way to reduce nitrogen loss. Biochar was deemed a potentially applicable material to reduce nitrogen leaching^10^, and biochar is considered effective and stable in the long term^11^. Biochar is a carbon-rich, porous, and highly aromatic material formed through the pyrolysis of organic biomass under oxygen-limited conditions at temperatures between 175 and 950 °C. Biochar has dual benefits in climate change mitigation, carbon sequestration, and positive soil amendment^12^. Also, biochar contains significant amounts of essential plant nutrients, including nitrogen, phosphorus, potassium, and calcium, making it valuable for agricultural fertilization^13^. The pyrolysis temperatures of the feedstocks significantly impact the physical and chemical properties of the resulting biochar^14,15^. Pyrolysis can markedly decrease the mobility of inorganic contaminants and effectively reduce or eliminate organic and biological pollutants, while substantially increasing nutrient concentrations compared to the original feedstock^13^. It also plays a crucial role in influencing the cation exchange capacity of biochar, where the cation exchange capacity declines with increasing pyrolysis temperature. This decline is attributed to the decrease in functional groups such as -COOH and -OH groups^16^. Additionally, due to the variety of surface functional groups, biochar can increase soil cation exchange capacity and anion exchange capacity, which may be why it can prevent nitrogen leaching^17,18^. Bamboo biochar at 900 °C has a NO_3_^−^ adsorption capability of about 1.2 mg g^−1^^19^. The ammonium and nitrate were successfully absorbed by biochar by 3.7% and 15.7%, respectively^20^. However, several investigations have revealed that biochar has limited or no adsorption capacity for nitrate^21^. The following mechanisms have been implicated in the adsorption of NH_4_^+^ onto biochar surfaces: Amides and amines are created when ammonium (NH_4_^+^) reacts with acidic functional groups^22^, attracting to negatively charged surfaces^23^, binding to cationic species locations on the biochar surfaces^24^; and electron donor-acceptor interactions^25^. Biochar applications decreased total cumulative losses of NH_4_^+^ through leaching by 15.2% at a depth of 20 cm^26^. The application of 20 g kg^−1^ biochar treatments lowered total nitrogen leached by 11% in the columns treated with manure^27^. When compared to other organic additions, biochar offers an alternate option to reduce agricultural waste in addition to its contribution to improving the physical and chemical qualities of soil. Biochar production greatly reduces the volume and weight of animal and plant waste and necessitates fewer applications than fertilizers, which must be applied on an annual basis^28^. Tomato is one of the most important crops in Qena Governorate, with a cultivated area of 33,900 feddans (14,238 hectares). Approximately 69,156 tons of tomato stems are generated in this area, constituting a significant agricultural residue and a potential environmental concern.

Although biochar has been widely studied as a soil amendment, clear knowledge gaps remain regarding the effects of tomato stems biochar produced at different pyrolysis temperatures on the leaching behavior of nitrate, ammonium, and dissolved organic carbon (DOC) in sandy soils. Current research provides only limited and contradictory insights into how biochar dose and pyrolysis temperature affect nitrate, ammonium, and DOC dynamics in soils. This study hypothesizes that biochar produced from tomato stems at varying temperatures and doses will significantly affect soil properties and reduce nutrient loss by leaching. To test this hypothesis, biochar produced at 250, 400, and 600 °C was applied at different doses to sandy soil. The objective is to evaluate the effects of pyrolysis temperature and dose of tomato stems biochar on soil physical and chemical properties and the leaching dynamics of nitrate, ammonium, and DOC in sandy soil. This research is novel in systematically assessing how pyrolysis temperature and application rate of tomato stems biochar influence nitrate and ammonium retention, DOC mobility, and soil quality indicators in sandy soils, offering actionable recommendations for improving biochar use in coarse-textured agricultural systems.

## Materials and methods

### Biochar production

Biochar was produced from tomato stems (TS) cut up less than 5 cm and oven dried (70˚C), converted into biochar through slow pyrolysis using a furnace under oxygen-limited conditions. The tank was filled with the feedstock materials and tightly closed. Three different pyrolysis temperatures, 250 °C for 10 h, 400 °C, and 600 ˚C for 4 h, all biochar samples were crushed and ground to pass through a 2-mm sieve for chemical analysis. Tomato stems biochar produced at 250 °C (TSB250), 400 °C (TSB400), and 600 °C (TSB600). The pH of biochar was measured in suspension ratio of 1:20 (w/v) by using a pH meter, while the electrical conductivity was measured in biochar extract ratio of 1:20 (w/v) with an EC meter^29^. The organic matter content in biochar was determined by the Walkley-Black method^30^. The total content of nitrogen, phosphorus, and potassium was obtained by digesting tomato stem biochar with concentrated sulfuric acid (H_2_SO_4_), hydrogen peroxide (H_2_O_2_), and salicylic acid (C_7_H_6_O_3_)^31^. Nitrogen content was determined by the Kjeldahl method^32^, phosphorus in the digests was analyzed photometrically by chlorostannous phosphomolybdic acid method^30^, and potassium in the digests was measured by flame photometer. Some chemical properties of tomato stems biochar (TSB) are listed in Table 1.

**Table 1:** Some important properties of the tomato stems biochar produced at different pyrolysis temperatures (Data were mean ± standard deviation, *n* = 3).

Property	Tomato stems biochar produced at 250 °C	Tomato stems biochar produced at 400 °C	Tomato stems biochar produced at 600 °C
pH	6.58 ± 0.01	8.38 ± 0.00	9.23 ± 0.01
EC (dS m^−1^)	6.91 ± 0.05	8.05 ± 0.02	8.26 ± 0.08
C (%)	39.21 ± 0.03	47.10 ± 0.07	50.30 ± 0.10
Total N (%)	0.54 ± 0.02	0.38 ± 0.05	0.29 ± 0.07
Total P (%)	0.19 ± 0.10	0.21 ± 0.08	0.28 ± 0.06
Total K (%)	0.26 ± 0.05	0.30 ± 0.05	0.33 ± 0.02
DOC (mg kg^−1^)	8450.04 ± 0.34	4200.13 ± 0.73	3500.02 ± 1.43
CEC (cmolc kg^−1^)	19.82 ± 0.01	38.51 ± 0.02	10.60 ± 0.01

*EC* electrical conductivity, *DOC* dissolved organic carbon, *CEC* cation exchange capacity.

### Design of column experiment

The column experiment was conducted in the laboratory of the Soils and Water Department, Faculty of Agriculture, South Valley University, Qena, Egypt. The sandy soil used in this experiment was collected from an uncultivated field in South Valley University at a depth of 0–30 cm. The site of soil sampling is located at latitude 26° 11’ 22.2’’ N and longitude 32° 44’ 25.5’’ E, and 81 m above sea level. The soil under study is classified as Entisols; Typic Torripsamments (U.S. Soil Taxonomy). The soil was air-dried and then sieved with a sieve (2 mm). The chemical and physical properties of tomato stems biochar (TSB) are listed in Table 2. The experiment was including these treatments; control (no biochar added), 1% TSB250, 2.5% TSB250, 5% TSB250, 1% TSB400, 2.5% TSB400, 5% TSB400, 1% TSB600, 2.5% TSB600, and 5% TSB600. Tomato stems biochar was applied at three doses 1%, 2.5%, and 5% (w/w). This experiment was placed in a completely randomized design with three replicates. The leaching experiment was carried out in a set of plastic columns with dimensions of a height of 40 cm and an inner diameter of 5.5 cm. The columns were provided with an end cap perforated with a piece of cloth at the bottom. Each column was filled with 1 kg of sandy soil. This experiment was conducted for seven weeks, with leaching performed once per week under laboratory conditions at room temperature. The average ambient air temperature remained relatively stable throughout the experimental period. The columns were leached in the first week by 110% of their water-holding capacity (WHC) with distilled water, and the weight of the column and the added water (primary weight). Then the leachate was received with a cup. The soil in the columns was leached to eliminate nitrate and ammonium. In the second week, ammonium nitrate fertilizer was added by a nutrient solution at a rate of 48.9 mg NH_4_/kg soil, and 26.7 mg NO_3_/kg soil, was prepared by dissolving ammonium nitrate (NH_4_NO_3_) in distilled water for each column that’s then the volume of the filtrate was measured and the weight of each column before adding water to reach the (primary weight) until a stable filtrate volume was obtained. Throughout the six-week experimental period, leachate from each column was collected in a 250 mL glass conical flask at each sampling time. The amounts of water added and leached during leaching cycles in the soil under study are presented in Table 3.

**Table 2:** Some selected properties of the tested soil under study (Data were mean ± standard deviation, *n* = 3).

Property	Value ± SD
Sand (%)	89
Silt (%)	6
Clay (%)	5
Texture	Sand
Organic matter (g kg^−1^)	4.61 ± 0.06
DOC (mg kg^−1^)	10.00 ± 0.06
EC_1:5_ (dS m^−1^)	0.50 ± 0.01
pH_1:2.5_	8.28 ± 0.02
CEC (cmolc kg^−1^)	3.50 ± 0.01
Available phosphorus (mg kg^−1^)	9.81 ± 0.53
Available potassium (mg kg^−1^)	36.03 ± 0.03

*DOC* dissolved organic carbon, *EC* electrical conductivity, *CEC* cation exchange capacity.

**Table 3:** Amounts of water added and leached during leaching cycles in the soil under study (Data were mean ± standard deviation, *n* = 3).

Treatment	1st leaching cycle (1 day)		2nd leaching cycle (7 days)		3rd leaching cycle (14 days)		4th leaching cycle (21 days)		5th leaching cycle (28 days)		6th leaching cycle (35 days)	
	Added water	leachate	Added water	leachate	Added water	leachate	Added water	Leachate	Added water	leachate	Added water	leachate
Control	242.00	30.00 ± 1.00	50.00	28.00 ± 0.00	55.00	33.00 ± 1.00	56.00	32.00 ± 2.00	55.40	32.50 ± 0.50	54.00	32.00 ± 1.00
TSB250-1%	253.00	30.00 ± 2.00	47.00	28.50 ± 0.50	47.00	28.50 ± 0.50	46.10	27.80 ± 1.30	45.60	26.50 ± 0.50	46.00	26.00 ± 0.00
TSB250-2.5%	275.00	48.00 ± 1.00	56.00	36.00 ± 1.00	55.00	34.50 ± 0.50	54.90	35.20 ± 1.30	54.40	33.20 ± 0.70	54.80	32.50 ± 0.90
TSB250-5%	297.00	32.00 ± 1.00	52.00	36.00 ± 0.00	53.00	36.00 ± 0.00	51.00	35.20 ± 0.70	50.50	33.60 ± 0.50	50.90	32.00 ± 0.00
TSB400-1%	258.50	28.00 ± 1.70	48.00	29.00 ± 1.00	49.00	29.00 ± 0.00	47.00	28.30 ± 0.60	46.50	27.00 ± 0.00	46.90	26.40 ± 0.50
TSB400-2.5%	302.50	45.60 ± 0.60	56.00	35.50 ± 0.50	58.00	36.50 ± 0.50	54.90	34.70 ± 0.60	54.40	33.00 ± 1.00	54.10	32.00 ± 1.00
TSB400-5%	330.00	42.60 ± 0.60	54.00	35.00 ± 1.00	55.00	35.00 ± 0.00	52.90	34.20 ± 0.70	52.40	32.50 ± 0.50	52.00	31.00 ± 0.00
TSB600-1%	264.00	29.00 ± 0.00	44.50	26.00 ± 2.60	47.00	27.50 ± 0.90	43.60	25.30 ± 0.60	43.20	24.20 ± 1.10	43.50	23.00 ± 1.00
TSB600-2.5%	319.00	47.80 ± 1.30	58.00	36.00 ± 2.60	59.00	37.50 ± 0.50	56.80	35.20 ± 0.30	56.20	33.50 ± 0.50	56.70	32.80 ± 0.30
TSB600-5%	363.00	45.00 ± 0.00	54.00	34.50 ± 0.90	56.00	35.80 ± 1.30	52.90	33.70 ± 0.50	52.30	32.00 ± 0.00	52.80	31.40 ± 0.50

TSB250: tomato stems biochar produced at 250 °C; TSB400: tomato stems biochar produced at 400 °C; TSB600: tomato stems biochar produced at 600 °C. Tomato stems biochar was applied at three doses 1%, 2.5%, and 5% (w/w).

### Soil analysis

The soil texture was determined by the hydrometer^33^. The pH of soil and soil–biochar mixtures was measured in a ratio of 1:2.5 (w/v) soil-to-distilled water suspension. Electrical conductivity (EC) was determined in a ratio of 1:5 (w/v) soil-to-distilled water extract. Cation exchange capacity (CEC) was determined using the ammonium acetate (NH_4_CH_3_COO) method^34^. Organic matter content in soil and biochar was determined by the Walkley-Black method, and calcium carbonate content in the soil samples was estimated using a Collins calcimeter^30^. The available nitrogen in the soil after the end of this experiment was extracted by a 2 M potassium chloride (KCl) solution, and available nitrogen (NH_4_^+^ and NO_3_^−^) content was determined by the Kjeldahl method^35^. Available phosphorus (Olsen-P) in soil, biochar, and their mixtures was extracted by 0.5 M sodium bicarbonate (NaHCO_3_) solution at pH 8.5^36^, spectrophotometrically determined^30^. The available potassium was determined by the NH_4_CH_3_COO extraction method (1 M, pH 7), and potassium was determined using the flame photometry method^30^, at a ratio of 1:20 (biochar: water) and a 1:5 ratio of soil to water distilled (w/v) in both. The nitrate and ammonium concentration in the leachate was determined by the Kjeldahl distillation unit^35^ and dissolved organic carbon (DOC)^37^.

### Statistical analyses

All data obtained were statistically analyzed using analysis of variance (ANOVA) by Statistix version 9.0. The differences among the treatments were analyzed by Tukey’s honestly significant difference test (Tukey’s HSD) at the 0.05 level of probability (p).

## Results

### Effects of biochar on some soil properties

**Fig. 1:**
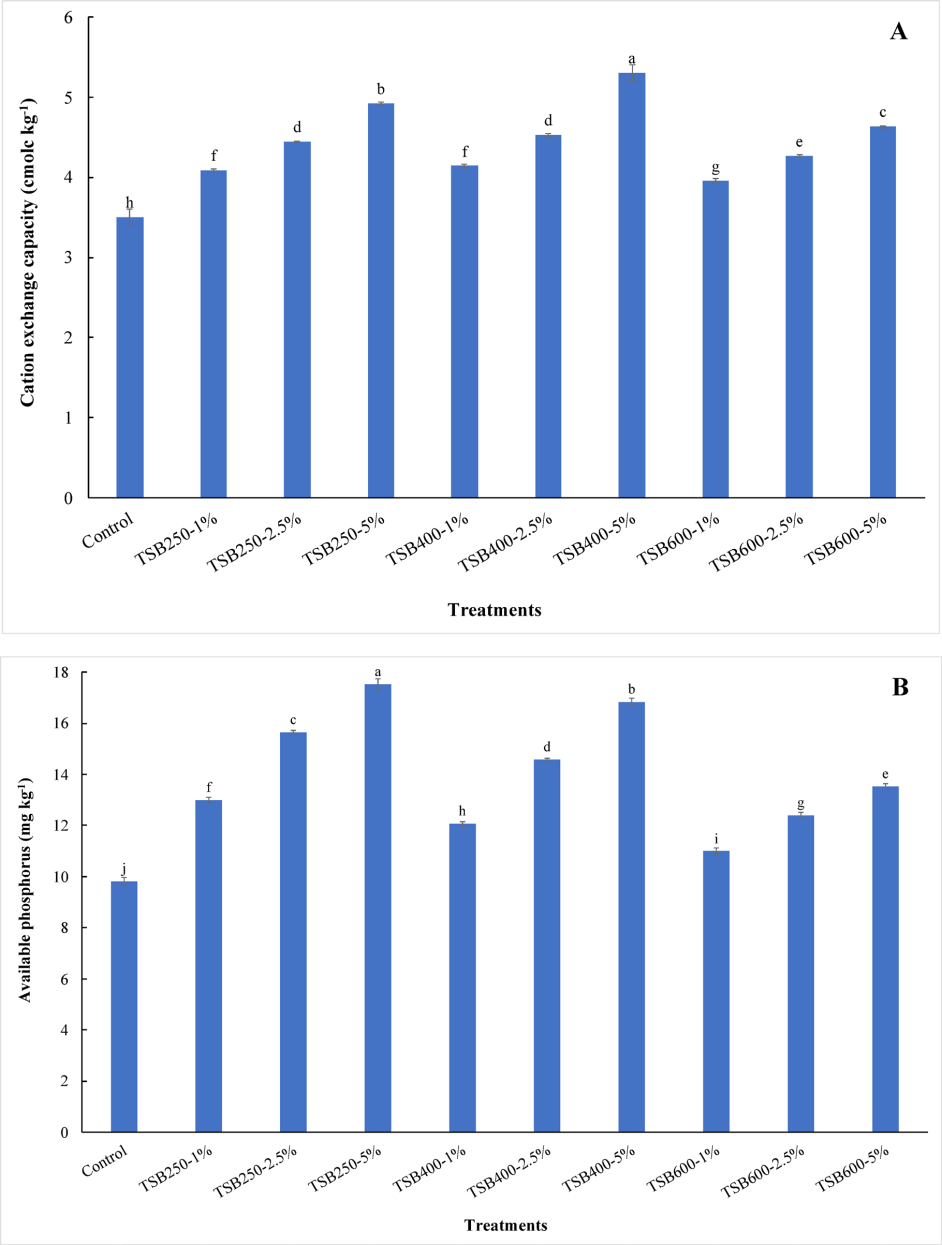
Variability in cation exchange capacity (**A**) and available phosphorus (**B**) in sandy soil as affected by pyrolysis temperatures and application doses of tomato stems biochar. TSB250: tomato stems biochar produced at 250 °C; TSB400: tomato stems biochar produced at 400 °C; TSB600: tomato stems biochar produced at 600 °C. Tomato stems biochar was applied at three doses 1%, 2.5%, and 5% (w/w). Different lowercase letters on each bar indicate the significant differences among treatments according to Tukey’s Honestly Significant Difference test at *p* ≤ 0.05. Vertical bars indicate the standard deviation of the mean (*n* = 3 replicates).

Water-holding capacity in sandy soil increased significantly (*p* ≤ 0.05) with the application of biochar at all pyrolysis temperatures and doses. The content of water holding capacity increased from 220.00 g kg^−1^ for control treatment to 230.00, 250.00, 270.00, 235.00, 275.00, 300.00, 240.00, 290.00, 330.00 g kg^−1^ for 1% TSB250, 2.5% TSB250, 5% TSB250, 1% TSB400, 2.5% TSB400, 5% TSB400, 1% TSB600, 2.5% TSB600, and 5% TSB600 treatments, respectively (Table 4). The content of water-holding capacity in sandy soil increased significantly with increasing pyrolysis temperatures and doses (Table 4). The results obtained from this study show that the biochar applications at all pyrolysis temperatures and levels significantly increased organic matter content in sandy soil compared to unamended soil. Organic matter content increased from 0.46 mg kg^−1^ (control) to 0.81, 1.28, 2.19, 0.98, 1.57, 2.84, 1.15, 2.08, and 3.39 mg kg^−1^ for 1% TSB250, 2.5% TSB250, 5% TSB250, 1% TSB400, 2.5% TSB400, 5% TSB400, 1% TSB600, 2.5% TSB600, and 5% TSB600 treatments, respectively (Table 4). Compared with the control, TSB250 applications at 2.5% and 5% significantly decreased soil pH (*p* ≤ 0.05), whereas all other treatments significantly increased soil pH (Table 4). The soil pH values increased with increasing pyrolysis temperature of biochar. The soil pH decreased from 8.27 for the control to 8.24 and 8.21 for 2.5% TSB250 and 5% TSB250 treatments, respectively (Table 4). Applying tomato stems biochar to sandy soil increased soil pH values from 8.27 for the control to 8.31, 8.33, 8.36, 8.41, 8.37, 8.43, and 8.46 for 1% TSB250, 1% TSB400, 2.5% TSB400, 5% TSB400, 1% TSB600, 2.5% TSB600, and 5% TSB600 treatments, respectively (Table 4). The highest pH value was observed at 5% TSB600 treatment. However, the lowest pH value was observed at 5% TSB250 treatment. Compared to the control, electrical conductivity (EC) in sandy soil increased significantly with adding TSB250-5%, TSB400-2.5%, TSB400-5%, TSB600-2.5%, and TSB600-5% treatments (Table 4). The values of EC increased from 0.50 dS m^−1^ for control treatment to 0.71, 0.72, 0.89, 0.64, 0.75, 1.28, 0.73, 0.87, and 1.23 dS m^−1^ for 1% TSB250, 2.5% TSB250, 5% TSB250, 1% TSB400, 2.5% TSB400, 5% TSB400, 1% TSB600, 2.5% TSB600, and 5% TSB600 treatments, respectively (Table 4). The results indicated that the highest value of EC was observed when applying 5% TSB400 to sandy soil. The applications of biochar at all pyrolysis temperatures and levels in sandy soil caused significant increases in the cation exchange capacity (CEC) compared to unamended soil. The highest value of CEC was observed at 5% TSB400 treatment (Fig. 1A). The CEC values increased from 3.50 cmolc kg^−1^ for control to 4.09, 4.44, 4.92, 4.14, 4.53, 5.30, 3.96, 4.27, and 4.63 cmolc kg^−1^ for 1% TSB250, 2.5% TSB250, 5% TSB250, 1% TSB400, 2.5% TSB400, 5% TSB400, 1% TSB600, 2.5% TSB600, and 5% TSB600 treatments, respectively. The effectiveness of biochar treatments in increasing CEC followed the order: TSB400 > TSB250 > TSB600 (Fig. 1A). All biochar treatments caused significantly increased Olsen-P in the soil under study compared to the control treatment. The concentrations of Olsen-P increased from 9.83 mg kg^−1^ (control) to 13.00, 15.65, 17.53, 12.07, 14.57, 16.84, 10.99, 12.41, and 13.53 mg kg^−1^ for 1% TSB250, 2.5% TSB250, 5% TSB250, 1% TSB400, 2.5% TSB400, 5% TSB400, 1% TSB600, 2.5% TSB600, and 5% TSB600 treatments, respectively (Fig. 1B). The concentrations of Olsen-P increased with increasing biochar levels. Therefore, the Olsen-P concentration decreased significantly with increasing pyrolysis temperature at the same biochar level (Fig. 1B). At the end of the leaching cycles, the available potassium concentration significantly increased when applying all biochar treatments compared to the control treatment. Available potassium concentration increased from 36.04 mg kg^−1^ for control to 324.62, 476.79, 689.82, 398.53, 581.13, 928.07, 372.44, 563.74, and 820.25 mg kg^−1^ for 1% TSB250, 2.5% TSB250, 5% TSB250, 1% TSB400, 2.5% TSB400, 5% TSB400, 1% TSB600, 2.5% TSB600, and 5% TSB600 treatments, respectively. The concentrations of available potassium increased with increasing biochar levels (Table 4).

**Table 4:** Effects of pyrolysis temperatures and doses of tomato stems biochar on changes in physical and chemical properties of sandy soil (Data were mean ± standard deviation, *n* = 3).

Treatments	WHC (g kg^−1^)	O.M (g kg^−1^)	pH	EC (dS m^−1^)	Available K (mg kg^−1^)
Control	220.00 ± 2.00^j^	0.46 ± 0.02^j^	8.27 ± 0.01^f^	0.50 ± 0.06^d^	36.04 ± 4.58^g^
TSB250-1%	230.00 ± 1.73^i^	0.81 ± 0.01^i^	8.31 ± 0.01^e^	0.71 ± 0.04^bcd^	324.62 ± 7.53^f^
TSB250-2.5%	250.00 ± 1.00^f^	1.28 ± 0.03^f^	8.24 ± 0.01^g^	0.72 ± 0.04^bcd^	476.79 ± 32.82^de^
TSB250-5%	270.00 ± 3.46^e^	2.19 ± 0.02^c^	8.21 ± 0.03^g^	0.89 ± 0.06^b^	689.82 ± 32.82^bc^
TSB400-1%	235.00 ± 2.65^h^	0.98 ± 0.01^h^	8.33 ± 0.01^de^	0.64 ± 0.01^cd^	398.53 ± 45.80^ef^
TSB400-2.5%	275.00 ± 1.00^d^	1.57 ± 0.04^e^	8.36 ± 0.01^cd^	0.75 ± 0.03^bc^	581.13 ± 91.61^cd^
TSB400-5%	300.00 ± 0.00^b^	2.84 ± 0.02^b^	8.41 ± 0.02^b^	1.28 ± 0.20^a^	928.07 ± 78.70^a^
TSB600-1%	240.00 ± 2.65^g^	1.15 ± 0.02^g^	8.37 ± 0.01^c^	0.73 ± 0.13^bcd^	372.44 ± 37.65^ef^
TSB600-2.5%	290.00 ± 1.73^c^	2.08 ± 0.03^d^	8.43 ± 0.01^b^	0.87 ± 0.05^bc^	563.74 ± 39.13^cd^
TSB600-5%	330.00 ± 2.00^a^	3.39 ± 0.03^a^	8.46 ± 01^a^	1.23 ± 0.05^a^	820.25 ± 52.72^ab^

TSB250: tomato stems biochar produced at 250 °C; TSB400: tomato stems biochar produced at 400 °C; TSB600: tomato stems biochar produced at 600 °C. Tomato stems biochar was applied at three doses 1%, 2.5%, and 5% (w/w). Different superscript lowercase letters in each column showed significant differences between treatments according to Tukey’s Honestly Significant Difference test at *P* ≤ 0.05.

### Leaching nitrogen from the sandy soil

#### Effects of biochar on leached ammonium and nitrate

In the first and second leaching cycles, the results obtained from this study show that the biochar applications at all pyrolysis temperatures and levels in the sandy soil caused significant decreases in the concentrations of leaching ammonium and nitrate compared to unamended soil. Moreover, the leaching of ammonium from the sandy soil decreased significantly with increasing biochar doses at all pyrolysis temperatures. However, nitrate leaching from sandy soil decreased significantly with increasing doses of TSB600 biochar (Table 5). The amount of leaching ammonium in the first leaching cycle decreased from 12.44 mg kg^−1^ (unamended soil) to 2.49, 1.71, 1.32, 1.90, 1.57, 1.21, 3.20, 2.56, and 2.12 mg kg^−1^ for 1% TSB250, 2.5% TSB250, 5% TSB250, 1% TSB400, 2.5% TSB400, 5% TSB400, 1% TSB600, 2.5% TSB600, and 5% TSB600 treatments, respectively (Table 5). In the second leaching cycle, the concentrations of leaching ammonium decreased from 8.24 mg kg^−1^ for control treatment to 4.99, 4.79, 4.30, 3.64, 3.60, 3.09, 4.05, 3.94, and 3.55 mg kg^−1^ for 1% TSB250, 2.5% TSB250, 5% TSB250, 1% TSB400, 2.5% TSB400, 5% TSB400, 1% TSB600, 2.5% TSB600, and 5% TSB600 treatments, respectively (Table 5). The concentration of leaching nitrate in the first leaching cycle decreased from 13.51 mg kg^−1^ (control) to 2.17, 2.16, 2.15, 2.08, 2.07, 2.07, 1.74, 1.32, 1.14 mg kg^−1^ for 1% TSB250, 2.5% TSB250, 5% TSB250, 1% TSB400, 2.5% TSB400, 5% TSB400, 1% TSB600, 2.5% TSB600, and 5% TSB600 treatments, respectively (Table 5). The concentration of leaching nitrate in the second leaching cycle decreased from 4.80 mg kg^−1^ (control) to 4.24, 4.22, 4.21, 4.24, 4.33, 4.32, 3.24, 3.17, and 3.08 mg kg^−1^ for 1% TSB250, 2.5% TSB250, 5% TSB250, 1% TSB400, 2.5% TSB400, 5% TSB400, 1% TSB600, 2.5% TSB600, and 5% TSB600 treatments, respectively (Table 5). On the other hand, leaching ammonium increased significantly with the biochar applications at all pyrolysis temperatures and levels in the sandy soil in comparison with the control treatment in the third, fourth, fifth, and sixth leaching cycles (Table 5).

**Table 5:** Effects of pyrolysis temperatures and doses of tomato stems biochar on leaching ammonium and nitrate in sandy soil (Data were mean ± standard deviation, *n* = 3).

Treatments	Leaching losses (mg kg^−1^)											
	1st leaching cycle 1 day		2nd leaching cycle 7 days		3rd leaching cycle 14 days		4th leaching cycle 21 days		5th leaching cycle 28 days		6th leaching cycle 35 days	
	NH_4_^+^-*N*	NO_3_^−^-*N*	NH_4_^+^-*N*	NO_3_^−^-*N*	NH_4_^+^-*N*	NO_3_^−^-*N*	NH_4_^+^-*N*	NO_3_^−^-*N*	NH_4_^+^-*N*	NO_3_^−^-*N*	NH_4_^+^-*N*	NO_3_^−^-*N*
Control	12.44 ± 0.05^a^	13.51 ± 0.01^a^	8.24 ± 0.01^a^	4.80 ± 0.01^a^	3.68 ± 0.01^h^	1.50 ± 0.08^f^	1.54 ± 0.05^j^	0.54 ± 0.02^f^	0.23 ± 0.03^h^	0.14 ± 0.05^f^	0.16 ± 0.04^g^	0.06 ± 0.01^d^
TSB250-1%	2.49 ± 0.04^c^	2.17 ± 0.06^c^	4.99 ± 0.11^b^	4.24 ± 0.06^c^	5.43 ± 0.09^a^	4.90 ± 0.05^ab^	4.38 ± 0.09^b^	5.00 ± 0.06^b^	2.83 ± 0.01^c^	2.29 ± 0.06^a^	0.71 ± 0.04^c^	0.25 ± 0.01^a^
TSB250-2.5%	1.71 ± 0.09^g^	2.16 ± 0.02^c^	4.79 ± 0.04^c^	4.22 ± 0.02^c^	5.21 ± 0.04^b^	4.89 ± 0.04^ab^	4.22 ± 0.05^d^	4.98 ± 0.01^b^	2.60 ± 0.19^d^	2.29 ± 0.02^a^	0.65 ± 0.01^d^	0.25 ± 0.01^a^
TSB250-5%	1.32 ± 0.01^i^	2.15 ± 0.04^c^	4.30 ± 0.01^d^	4.21 ± 0.03^c^	4.47 ± 0.01^d^	4.88 ± 0.06^ab^	3.81 ± 0.01^f^	4.95 ± 0.03^b^	2.11 ± 0.02^g^	2.28 ± 0.03^a^	0.53 ± 0.04^f^	0.25 ± 0.02^a^
TSB400-1%	1.90 ± 0.05^d^	2.08 ± 0.01^d^	3.64 ± 0.05 ^g^	4.24 ± 0.05^c^	4.34 ± 0.03^e^	4.91 ± 0.04^a^	3.99 ± 0.15^e^	5.10 ± 0.09^a^	2.77 ± 0.04^c^	2.21 ± 0.02^b^	0.69 ± 0.05^cd^	0.25 ± 0.03^a^
TSB400-2.5%	1.57 ± 0.04^f^	2.07 ± 0.03^d^	3.60 ± 0.04^g^	4.33 ± 0.21^b^	3.76 ± 0.04^g^	4.88 ± 0.01^b^	3.76 ± 0.03^f^	5.09 ± 0.02^a^	2.37 ± 0.03^f^	2.21 ± 0.04^b^	0.59 ± 0.04^e^	0.25 ± 0.01^a^
TSB400-5%	1.21 ± 0.04^h^	2.07 ± 0.01^d^	3.09 ± 0.01^h^	4.32 ± 0.03^b^	3.37 ± 0.02^i^	4.89 ± 0.11^ab^	3.28 ± 0.09^h^	5.06 ± 0.01^a^	2.05 ± 0.01^g^	2.19 ± 0.02^b^	0.51 ± 0.04^f^	0.24 ± 0.01^a^
TSB600-1%	3.20 ± 0.10^b^	1.74 ± 0.07^b^	4.05 ± 0.05^e^	3.24 ± 0.01^d^	5.11 ± 0.01^c^	3.64 ± 0.01^c^	4.88 ± 0.01^a^	3.90 ± 0.01^c^	3.40 ± 0.04^a^	1.98 ± 0.01^c^	0.85 ± 0.03^a^	0.22 ± 0.02^b^
TSB600-2.5%	2.56 ± 0.04^c^	1.32 ± 0.04^e^	3.94 ± 0.06^f^	3.17 ± 0.05^e^	4.52 ± 0.11^d^	3.60 ± 0.01^d^	4.31 ± 0.07^c^	3.83 ± 0.01^d^	3.46 ± 0.08^a^	1.91 ± 0.02^d^	0.86 ± 0.03^a^	0.21 ± 0.01^b^
TSB600-5%	2.12 ± 0.05^e^	1.14 ± 0.03^f^	3.55 ± 0.09^i^	3.08 ± 0.03^f^	4.08 ± 0.02^f^	3.53 ± 0.01^e^	3.98 ± 0.02^e^	3.77 ± 0.08^e^	3.12 ± 0.06^b^	1.77 ± 0.02^e^	0.78 ± 0.01^b^	0.19 ± 0.01^c^

TSB250: tomato stems biochar produced at 250 °C; TSB400: tomato stems biochar produced at 400 °C; TSB600: tomato stems biochar produced at 600 °C. Tomato stems biochar was applied at three doses 1%, 2.5%, and 5% (w/w). Different superscript lowercase letters in each column showed significant differences between treatments according to Tukey’s Honestly Significant Difference test at *P* ≤ 0.05.

#### Effects of biochar on cumulative leached ammonium and nitrate

**Fig. 2:**
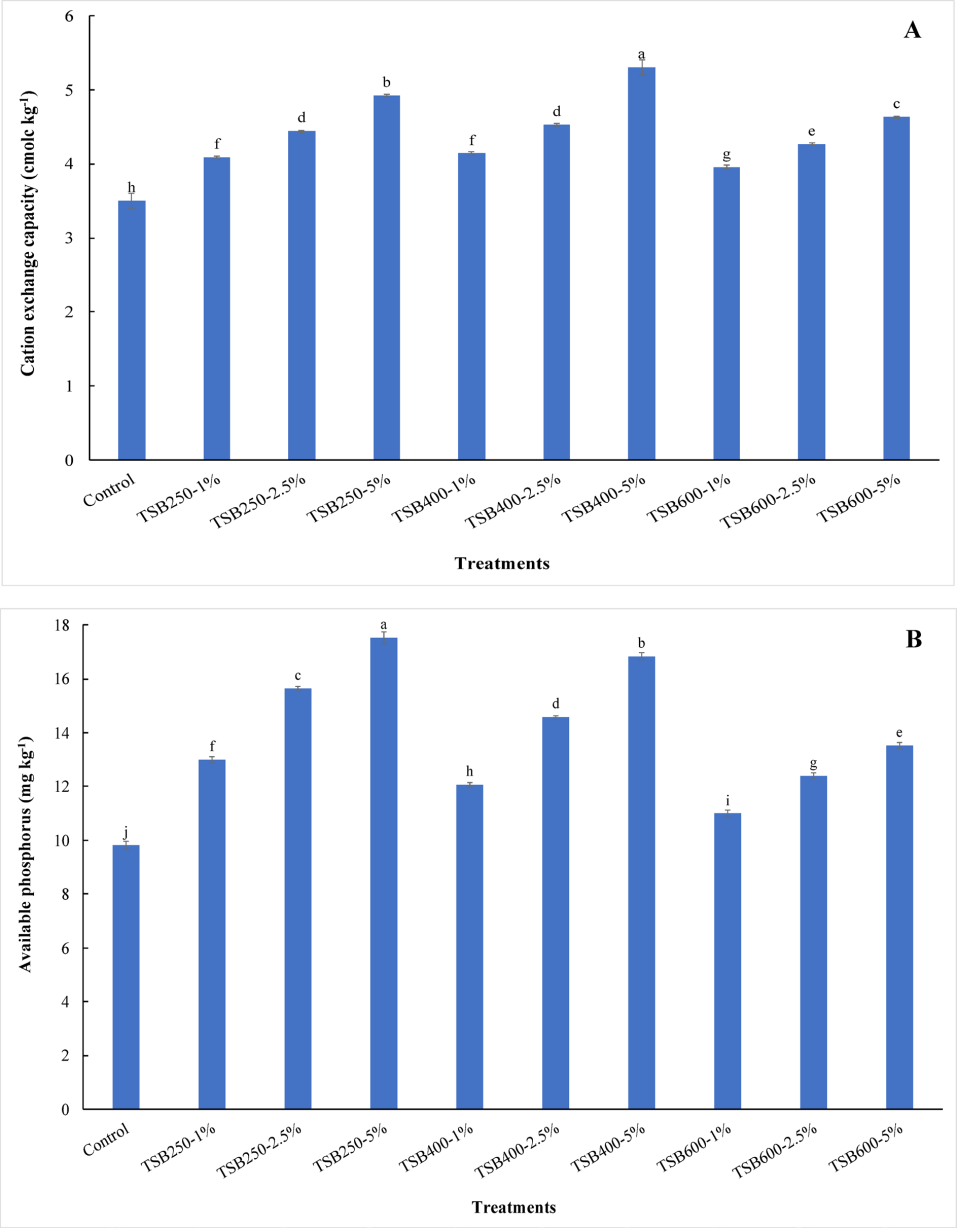
Effect of pyrolysis temperatures and application doses of tomato stems biochar on cumulative leached ammonium and nitrate (**A**) and total nitrogen (**B**) in sandy soil. TSB250: tomato stems biochar produced at 250 °C; TSB400: tomato stems biochar produced at 400 °C; TSB600: tomato stems biochar produced at 600 °C. Tomato stems biochar was applied at three doses 1%, 2.5%, and 5% (w/w). Different lowercase letters on each bar indicate the significant differences among treatments according to Tukey’s Honestly Significant Difference test at *p* ≤ 0.05. Vertical bars indicate the standard deviation of the mean (*n* = 3 replicates).

The concentration of cumulative leached ammonium and nitrate from sandy soil decreased significantly under applications of tomato stems biochar at all pyrolysis temperatures and levels compared to the control treatment (Fig. 2A). Cumulative leaching ammonium decreased from 26.29 mg kg^−1^ for unamended soil to 20.83, 19.18, 16.54, 17.34, 15.66, 13.51, 21.49, 19.65, and 17.63 mg kg^−1^ for 1% TSB250, 2.5% TSB250, 5% TSB250, 1% TSB400, 2.5% TSB400, 5% TSB400, 1% TSB600, 2.5% TSB600, and 5% TSB600 treatments, respectively (Fig. 2A). The effectiveness of biochar treatments on the cumulative leaching of ammonium decreased in the order of TSB400 > TSB250 > TSB600. Cumulative leaching nitrate decreased from 20.55 mg kg^−1^ for unamended soil to 18.85, 18.79, 18.72, 18.78, 18.82, 18.77, 14.72, 14.05, and 13.48 mg kg^−1^ for 1% TSB250, 2.5% TSB250, 5% TSB250, 1% TSB400, 2.5% TSB400, 5% TSB400, 1% TSB600, 2.5% TSB600, and 5% TSB600 treatments, respectively. The effectiveness of biochar treatments on the cumulative leaching nitrate was in the order of TSB600 > TSB400 ≈ TSB250 (Fig. 2A).

#### Effects of biochar on cumulative leached total nitrogen

In the current study, the applications of tomato stems biochar produced at all pyrolysis temperatures and levels in sandy soil caused a significant reduce cumulative leaching nitrogen compared to unamended soil (Fig. 2B). The results indicated nitrogen leaching decreased significantly with increase pyrolysis temperature and increase the addition doses compared to the control treatment, cumulative total N leached decreased from 46.84 mg kg^−1^ for control to 39.68, 37.97, 35.26, 36.12, 34.48, 32.28, 36.21,33.70 and 31.11 mg kg^−1^ for 1% TSB250, 2.5% TSB250, 5% TSB250, 1% TSB400, 2.5% TSB400, 5% TSB400, 1% TSB600, 2.5% TSB600, and 5% TSB600 treatments, respectively (Fig. 2B). The lowest concentration of cumulative leaching nitrogen was observed by applying 5% TSB600. The effectiveness of biochar treatments on the cumulative leaching nitrogen decrease was in the order of TSB600 > TSB400 > TSB250 (Fig. 2B).

### Effects of biochar on available nitrogen

**Fig. 3:**
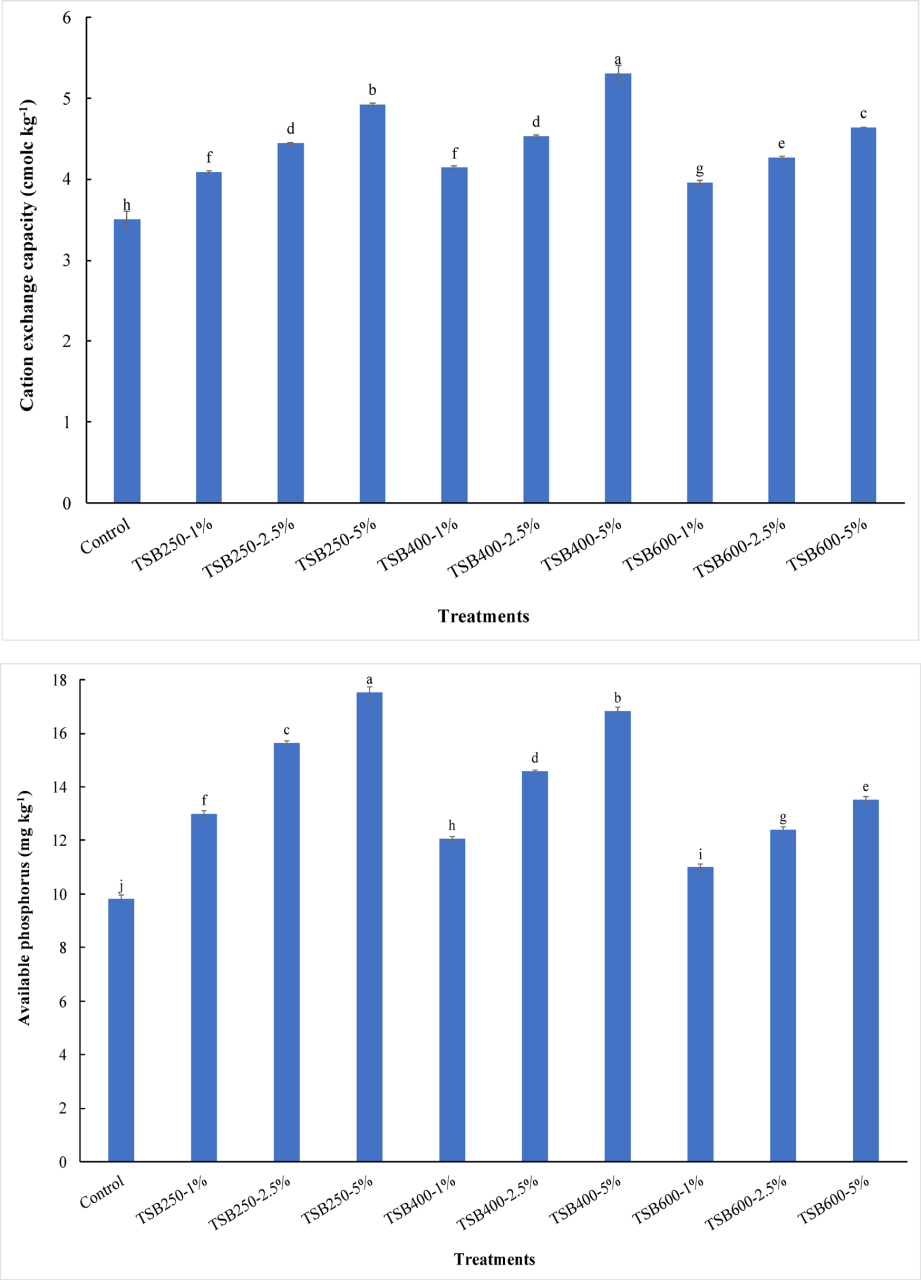
Changes in available nitrogen (**A**) and cumulative leaching of dissolved organic carbon (DOC) (**B**) in sandy soil as affected by pyrolysis temperatures and application doses of tomato stems biochar. TSB250: tomato stems biochar produced at 250 °C; TSB400: tomato stems biochar produced at 400 °C; TSB600: tomato stems biochar produced at 600 °C. Tomato stems biochar was applied at three doses 1%, 2.5%, and 5% (w/w). Different lowercase letters on each bar indicate the significant differences among treatments according to Tukey’s Honestly Significant Difference test at *p* ≤ 0.05. Vertical bars indicate the standard deviation of the mean (*n* = 3 replicates).

Compared to the control treatment, the applications of 1% TSB250 as well as TSB400 and TSB600 at all levels caused a significant increase in soil available nitrogen (*p* ≤ 0.05), but the rest of the treatments led to a significant decrease (Fig. 3A). The concentration of available nitrogen increased from 22.31 mg kg^−1^ for the control treatment to 24.47, 29.38, 32.73, 37.72, 25.71, 30.66, and 39.17 mg kg^−1^ for 1% TSB250, 1% TSB400, 2.5% TSB400, 5% TSB400, 1% TSB600, 2.5% TSB600, and 5% TSB600 treatments, respectively (Fig. 3A). On the other hand, the concentration of available nitrogen decreased from 22.31 mg kg^−1^ for the control treatment to 17.44 and 11.48 mg kg^−1^ for 2.5% TSB250 and 5% TSB250 treatments, respectively. The lowest concentration of available nitrogen was observed by applying 5% TSB250. The highest concentration of available nitrogen was observed by applying 5% TSB600 (Fig. 3A).

### Effect of biochar on leached dissolved organic carbon

In the first four leaching cycles, the results obtained from this study show that the biochar applications at all pyrolysis temperatures and levels in the sandy soil caused significant increases in the concentrations of leaching dissolved organic carbon compared to unamended soil. Moreover, the leaching of dissolved organic carbon in sandy soil increased significantly with increasing biochar doses (Table 6). In the first leaching, dissolved organic carbon content increased from 0.60 mg kg^−1^ for unamended soil to 29.14, 50.55, 93.71, 7.40, 10.61, 11.00, 5.70, 6.90, and 7.70 mg kg^−1^ for 1% TSB250, 2.5% TSB250, 5% TSB250, 1% TSB400, 2.5% TSB400, 5% TSB400, 1% TSB600, 2.5% TSB600, and 5% TSB600 treatments, respectively (Table 6). Therefore, the dissolved organic carbon concentration decreased significantly with increasing pyrolysis temperature. In the fifth leaching cycle, the addition of TSB250 at all levels and 5% TSB400 in the sandy soil caused significant increases in the concentrations of leaching dissolved organic carbon compared to unamended soil. In the sixth leaching cycle, the addition of TSB250 at all levels and TSB400 at 2.5 and 5% in the sandy soil caused significant increases in the concentrations of leaching dissolved organic carbon compared to unamended soil (Table 6).

**Table 6:** Effect of pyrolysis temperatures and doses of tomato stems biochar on leaching dissolved organic carbon (DOC) in sandy soil (Data were mean ± standard deviation, *n* = 3).

Treatments	DOC (mg kg^−1^)					
	1st leaching cycle	2nd leaching cycle	3rd leaching cycle	4th leaching cycle	5th leaching cycle	6th leaching cycle
Control	0.60 ± 0.00^g^	0.51 ± 0.08^g^	0.40 ± 0.04^e^	0.34 ± 0.05^h^	0.20 ± 0.00^d^	0.20 ± 0.01^e^
TSB250-1%	29.14 ± 0.47^c^	13.00 ± 0.46^c^	5.18 ± 0.28^c^	2.34 ± 0.43^c^	1.74 ± 0.13^bc^	1.64 ± 0.10^b^
TSB250-2.5%	50.55 ± 0.46^b^	23.50 ± 0.50^b^	6.67 ± 0.29^b^	3.60 ± 0.40^b^	2.00 ± 0.26^b^	1.90 ± 0.15^b^
TSB250-5%	93.71 ± 1.14^a^	45.30 ± 0.40^a^	10.00 ± 0.46^a^	5.50 ± 0.53^a^	3.01 ± 0.32^a^	2.80 ± 0.16^a^
TSB400-1%	7.40 ± 0.46^e^	3.00 ± 0.40^ef^	1.50 ± 0.35^cde^	0.90 ± 0.12^g^	1.10 ± 0.34^cd^	0.93 ± 0.21^cde^
TSB400-2.5%	10.61 ± 0.23^d^	4.80 ± 0.44^d^	2.81 ± 0.45^cd^	1.70 ± 008^de^	1.20 ± 0.06^bcd^	1.00 ± 0.18^bcd^
TSB400-5%	11.00 ± 0.40^d^	5.03 ± 0.35^d^	3.60 ± 0.53^bc^	1.80 ± 0.20^d^	1.30 ± 0.12^bc^	1.20 ± 0.22^bcd^
TSB600-1%	5.70 ± 0.21^f^	2.30 ± 0.82^f^	1.10 ± 0.15^de^	1.04 ± 0.13^fg^	0.70 ± 0.04^cd^	0.60 ± 0.07^de^
TSB600-2.5%	6.90 ± 0.45^ef^	2.80 ± 0.36^ef^	1.80 ± 0.26^cde^	1.21 ± 0.03^fg^	0.73 ± 0.03^cd^	0.65 ± 0.05^de^
TSB600-5%	7.70 ± 0.36^e^	3.37 ± 0.38^e^	2.80 ± 0.36^cd^	1.30 ± 0.13^ef^	0.74 ± 0.01^cd^	0.68 ± 0.03^de^

TSB250: tomato stems biochar produced at 250 °C; TSB400: tomato stems biochar produced at 400 °C; TSB600: tomato stems biochar produced at 600 °C. Tomato stems biochar was applied at three doses 1%, 2.5%, and 5% (w/w). Different superscript lowercase letters in each column showed significant differences between treatments according to Tukey’s Honestly Significant Difference test at *P* ≤ 0.05.

#### Effects of biochar on cumulative leaching of dissolved organic carbon

Applying tomato stems biochar produced at all pyrolysis temperatures and levels in sandy soil caused a significant increment in the cumulative leaching of dissolved organic carbon compared to the control treatment (Fig. 3B). The results indicated leaching dissolved organic carbon decreased significantly with increasing pyrolysis temperature and increased significantly with increasing doses compared to the control treatment, cumulative dissolved organic carbon leached increased from 2.24 mg kg^−1^ for control to 53.03, 88.22, 160.32, 14.82, 22.12, 23.94, 11.44, 14.08 and 16.58 mg kg^−1^ for 1% TSB250, 2.5% TSB250, 5% TSB250, 1% TSB400, 2.5% TSB400, 5% TSB400, 1% TSB600, 2.5% TSB600, and 5% TSB600 treatments, respectively (Fig. 3B). The lowest concentration of cumulative leaching dissolved organic carbon was observed in unamended soil. The effectiveness of biochar treatments on the cumulative leaching dissolved organic carbon increase was in the order of TSB250 > TSB400 > TSB600 (Fig. 3B).

## Discussion

The applications of biochar to sandy soils increased water retention compared to the unamended soil as well as it increased with increasing biochar doses^38^, the increased water retention in soil resulting from the presence of oxygen-containing functional groups on biochar surfaces, which would lead to the biochar surfaces being less hydrophobic^39^, increased total porosity, aggregation and structure of the soil, and high internal surface area^38,40^. On the other hand, water-holding capacity in amending soil with biochar decreased with increasing pyrolysis temperature^39^. Many studies found that the application of biochar produced at low-temperature pyrolysis to soils resulted in lower soil pH^41,42^. The decrease in soil pH is attributed to increasing acidic functional groups present on the surface of the biochar and producing organic acids and phenolic substances from cellulose during the decomposition process produced at low-temperature pyrolysis^43^. Increasing biochar doses led to a decrease in soil pH^44^. This explains the reason for the decreased pH soil value in treatments of TSB250 at 2.5 and 5% compared to the control. On the other hand, biochar applications in the soil increased pH and CEC. The results were similar to previous studies^45,46^. The pH and CEC of the resulting biochar are significantly influenced by the pyrolysis temperature and feedstock types^47,48^. Several studies found that the biochar produced at high pyrolysis temperatures was more alkaline, which is mainly attributed to an increase in the alkali cations such as Ca^2+^, Mg^2+^, K^+^, and Na^+^ as well as aromatic basal planes these ions increased the base saturation of the soil and reduced the proportion of exchangeable H^+^ in the soil, caused increased soil pH^49,50^. The high CEC of biochar-amended soils may be another factor contributing to a rise in soil pH^51^. Biochar’s ability to influence soil cation exchange capacity is dependent on feedstock type, pyrolysis temperature, application rate, initial soil CEC, and soil organic matter concentration^47,48^. The results obtained from this study were compatible with those of many researchers who found that treating soils with different levels of biochar produced at different pyrolysis temperatures led to increasing CEC values compared to the unamended soil^41,52^. This is due to the presence of acidic functional groups on biochar surfaces, which oxidize and cause an increase in negative charges, such as phenolic, carboxyl, and hydroxyl groups^53,54^, as well as biochar’s high CEC due to its high specific surface area and high porosity^55^. The results also showed a gradual increase in the cation exchange capacity of treatments BC400°C > BC250°C > BC600°C. These results are consistent with Yuan et al.^56^, who found that the biochar produced at 500 °C had significantly higher CEC than biochar produced at 300–700 °C. A positive relationship between biochar application and increased CEC in sandy soil has been reported; however, CEC values were observed to decline when biochar was produced at higher pyrolysis temperatures^39^. Has been attributed that CEC declined with increasing pyrolysis temperature to the decrease in some acid functional groups, such as carboxylic and hydroxyl groups^16^. Also, the functional groups in biochar, such as aliphatic, amide, aromatic amines, and aliphatic decreased as the pyrolysis temperature increased, while the stable aromatic structure increased^57^. The values of CEC in the soil increased with increasing biochar levels^42^. Previous studies found that the addition of biochar to alkaline sandy soils increased the amount of organic matter significantly with increasing biochar doses^58,59^. This increase in organic matter content in soils is attributed to the biochar-containing a high organic carbon content. Adding biochar to the alkaline sandy soils significantly improved the concentration of available potassium, which increased with increasing biochar doses. This increase in available potassium content in soils is attributed to the biochar-containing high potassium content^44,60^. The soluble content of potassium in sandy soil increased with increasing pyrolysis temperatures of applied biochar^41^. Applying biochar to alkaline sandy soil significantly increased available phosphorus, and it increased with increasing biochar doses^44^. This is attributed to biochar containing substantial amounts of phosphorus and can therefore directly contribute phosphorus to the soil solution, enhancing its availability in soil^16^. Biochar produced at lower pyrolysis temperatures shows greater potential to enhance the availability of applied phosphorus in alkaline soils, likely due to their lower pH, higher labile organic carbon content, and greater surface negative charge^61^.

The effect of biochar application on nitrogen leaching in the soils can be explained by four main mechanisms: (1) adsorbed chemically (chemical reaction with surface functional groups) (2), physical absorption (entrapped in the solution present in interior pores), (3) adsorbed electrostatically (cation or anion exchange to charged functional groups on biochar surfaces), and (4) mineralization and immobilization processes^62^. It is well expected that biochar may be a potential amendment to soils for nutrient retention due to its adsorption properties^63^. Enhancement of N retention in the soils treated by biochar has been reported^62^. Our results showed that the cumulative leached ammonium under all the biochar treatments was lower compared to the control. This confirms the effect of biochar on reducing leaching ammonium in the soil columns, which agrees with the results of previous studies^18,64^. This is mainly attributed to the capacity of biochar to absorb ammonium through cation exchange on negatively charged biochar functional groups^21^. The surface groups of biochar may be more essential than their surface area and porosity^22,65^. Biochar with high surface area does not have a higher ammonium adsorption capacity than biochar with low surface area, suggesting that surface area is not the most important factor influencing char^66^. This study shows that physisorption might not be the dominant mechanism for ammonium adsorption^66,67^. The increase in the CEC value of the soil caused by the biochar application might have been the main factor responsible for the observed reduction in ammonium leaching, as suggested by previous studies^18,68^.

Consistent with previous studies showing the variation in the effect of biochar on reducing nitrate leaching in sandy soil, amended with biochar from 5% to 37%^20,26,69^. This is consistent with our results, which showed the positive effect of adding biochar to the sandy soil in the columns experiment, as the cumulative leached nitrate decreased from 8.27% to 34.40% under tomato stems biochar treatments. A previous study also showed that the effect of biochar on NO_3_^−^ adsorption depends on many factors, the most important of which are the feedstock materials of biochar and the temperature of pyrolysis^70^. The sorption capacity of NO_3_^−^ of biochar produced at a high temperature (≥ 600 °C) increases, because these have a high surface area due to the formation of greater numbers of micropores and charge density^53,71^. Lawrinenko and Laird^72^ reported that the anion exchange capacity of biochar increases with increasing temperature of pyrolysis and oxonium positive surface groups, which increases binding capacity between negatively charged nitrates and some functional groups (oxonium) or positively charged salts on the surface of biochar^53,73^. Our results showed that the TSB600 treatments were the lowest in the cumulative leached nitrate, inversely, the TSB250 and TSB400 treatments show slight differences compared to the control. These differences also disappeared at the end of the leaching experiment. It is attributed to a decrease or no ability for NO_3_^−^ sorption found in biochar products at low temperatures^21,24^. Kameyama et al.^69^ found that NO_3_^−^ adsorbed chemically onto a functional group that is created at high temperatures^74^, and thus this is not a result of physical absorption. On the other hand, it can be explained by the increase in the leaching of nitrates during the first 21 days of the experiment and then the discouragement of nitrates leaching until the end of the experiment, the biochar application increased soil pH, improved soil air condition, and enhanced soil water holding capacity. Thus, this improved the biological activity and abundance of ammonia-oxidizing functional microorganisms in the soil and accelerated the soil nitrification rate^75,76^. In the end, the results showed the nitrate leaching amount decreased with increasing levels of biochar addition, which is consistent with the results of a previous study^77^. This reduction in nitrate leaching has been due to the nitrate adsorption capability of biochar^67^, and the probable mechanisms: (1) physical absorption as a secondary factor (solution flows into the biochar particles, where hydrated asymmetric nitrate ions are physically entrapped within the biochar pores^62,69^, and (2) adsorbed electrostatically as a major factor (bonding occurs between negatively charged nitrate and some functional groups such as (oxonium) or positively charged salts on the biochar surface^53,73^.

At the time of first application, tomato stems biochar resulted in significantly larger DOC losses in soil leachate than the control soils. Low-molecular-weight organic molecules that are labile or leachable, and some of which can adsorb to the surface of the biochar, are formed during the pyrolysis process^78^. The substantial losses from tomato stems biochar treatments after the initial application are most likely due to the biochar’s more labile polysaccharide organic matter, which does not adsorb effectively and is flushed into the DOM^79^. Discovered a similar impact when biochar was applied to sandy soils, the C source in DOC losses was largely biochar-derived rather than soil-derived, as well as DOC losses from biochar-augmented soils reduced with time, meaning that the more labile biochar-C was rapidly depleted^80^. All biochar treatments showed a higher cumulative loss of DOC compared with the control and it was arranged according to the cumulative loss of DOC: TSB250 > TSB400 > TSB600. These results are attributed to the DOC contents of biochar decreasing with increasing pyrolysis temperatures, which were similar results to some previous studies^10,15^. This might be attributed to the fact that increasing the pyrolysis temperature leads to an increase in aromatic carbon in the biochar, and non-aromatic carbon is reduced^81,82^. Probably the higher non-aromatic part in low-temperature biochar may make it easier for microbial bioactivity compared to high-temperature biochar^46^. Tomato stems biochar produced at lower temperatures may have stimulated microbial bioactivity and increased decomposition of soil organic matter, leading to higher DOC leaching than the high temperature biochar^81,83^. On the other hand, found decreased cumulative DOC leaching with the increase in pyrolytic temperatures, which is similar to our results and their explanation for this was that the increase in pyrolytic temperature increases the specific surface area, the micro-porosity, and functional groups, hence, increased DOC adsorption by biochar^29^.

## Conclusions

Excessive and continuous use of chemical nitrogen fertilizers in modern agriculture leads to increased pollution of the environment and groundwater. This study showed that the pyrolysis temperatures significantly affected the characteristics of biochar derived from tomato stems. The important physical and chemical properties of the sandy soil are potentially affected by adding tomato stems biochar produced at different pyrolysis temperatures. The results obtained from this study revealed the biochar’s potential to improve soil quality, such as water-holding capacity, cation exchange capacity, and nutrient availability, as well as decrease leaching of ammonium and nitrate in sandy soil. Using tomato stems biochar as a soil amendment is a promising method for greatly improving the quality indicators of sandy soil and reducing the leaching of ammonium and nitrate. Therefore, tomato stems biochar produced at different pyrolysis temperatures plays an important role in effectively improving nutrient retention in sandy soil. Field experiments should be carried out in future studies to ensure the efficiency of biochar use in sustainable agriculture. Overall, the application of biochar offers a viable pathway toward achieving a circular economy in agriculture by maximizing nutrient recycling, increasing nutrient use efficiency, and supporting long-term environmental and soil sustainability.

## Data Availability

The datasets used or analyzed during the current study are available from the corresponding author upon reasonable request.
